# Application of a maxillary transfacial approach to the caudal oral cavity and orbitozygomaticomaxillary complex in dogs

**DOI:** 10.3389/fvets.2023.1323983

**Published:** 2023-11-30

**Authors:** Graham P. Thatcher, Michael C. Congiusta, Jason W. Soukup

**Affiliations:** Dentistry and Oromaxillofacial Surgery, Department of Surgical Sciences, School of Veterinary Medicine, University of Wisconsin-Madison, WI, United States

**Keywords:** Weber–Ferguson approach, maxillary transfacial approach, dog, orbitozygomaticomaxillary complex, oral tumor

## Abstract

Surgical access and visualization for excision of large pathologic lesions of the orbitozygomaticomaxillary complex (OZMC) and caudal oral cavity can be a challenge in veterinary oromaxillofacial surgery and may limit one’s ability to perform such procedures. Combined intra- and extra-oral approaches as well as commissurotomy have been advocated in the past. However, each of these approaches present unique limitations specific to each approach. A transfacial approach that ameliorated these limitations could be advantageous. In this descriptive cohort study, we investigate the application and outcomes of a maxillary transfacial approach to the OZMC and caudal oral cavity in six client-owned dogs. The approach is based on the Weber–Ferguson approach for human applications and provided excellent exposure of the intended region in all six patients. We contend the maxillary transfacial approach provides some advantages to the more conventional combined intra-oral/extra-oral approach or commissurotomy for excision of large pathologic lesions of the OZMC and caudal oral cavity.

## Introduction

Primary goals of veterinary oncologic surgery are to achieve tumor-free margins while also maintaining good function and acceptable cosmetic outcome. Functional results are of particular importance with respect to oral tumor excisions in the caudal maxilla that may have deleterious impacts on the masticatory function and nasal airway patency. To increase exposure to the caudal oral cavity, including the caudal maxilla, palate and orbitozygomaticomaxillary complex (OZMC), combined intra-oral and extra-oral surgical approaches have been described (1–3). Although previous authors have reported improved surgical visualization and access to vital structures with combined intra-oral and extra-oral approaches, in the authors’ experience, this may require frequent repositioning of the patient. Access to caudal maxillary tumors have been enhanced by including a commissurotomy/buccotomy incision (3, 4). Although a commissurotomy does allow for greater access to the caudal maxilla, this incision alone is of minimal benefit if there is extensive involvement of the OZMC. Furthermore, a commissurotomy and buccotomy is likely to result in damage to the angularis oris cutaneous artery, which may be necessary for future soft tissue reconstruction efforts.

Since its introduction 150 years ago, the Weber–Ferguson approach to the midface has become the approach of choice to expose the maxilla, retromaxillary region and orbit in human patients. As described in humans, the approach begins with a lateral facial incision that extends in the anterior and inferior direction around the nasal ala to the philtrum and extended to the lip on midline. The oral incision is made along the gingivobuccal sulcus, extending caudally to the retromolar area. The resultant skin flap is elevated from the level of the periosteum and extended up to and along the lateral buttress of the maxilla. If exposure of the superior and lateral walls of the maxilla are required, the infraorbital neurovascular bundle is ligated and transected as the soft tissues of the cheek are raised from the anterior surface of the maxilla. Several modifications to the Weber–Ferguson approach, which provide enhanced surgical exposure to specific anatomical sites, have also been described for human patients (5–9). The Weber–Ferguson flap as applied to dogs and cats was first described by Asano (10). Purported benefits include improved exposure and angle of approach to osteotomies for caudal maxillary tumor excisions.

The Weber–Ferguson approach and the subsequent modifications, as has historically been true in human medicine, were named for the person(s) that originally developed and described them (11, 12). Veterinary medicine has a long tradition of not naming techniques, instruments, etc. after people. Rather, we use anatomical descriptions when possible. Therefore, we recommend use of the term *maxillary transfacial approach* for future descriptions of this approach in the veterinary literature.

In this descriptive study, we investigate the application of the maxillary transfacial approach (with appropriate extensions) to the caudal maxilla and retromaxillary region and report surgical outcomes in four dogs. In addition, we describe a modification applicable to dogs (*modified maxillary transfacial approach*). We contend this approach is an advantageous alternative to the combined intra-oral/extra-oral approach, as well as the commissurotomy, for excellent exposure for large tumors of the caudal maxilla and retromaxillary region in dogs that also spares the angularis oris artery.

## Materials and methods

### Case inclusion

Six client-owned dogs were presented to the Dentistry and Oromaxillofacial Surgery Service at the University of Wisconsin-Madison Veterinary Medical Center for assessment and surgical excision of tumors affecting the caudal maxilla and OZMC. Patients were between 7 and 13 years old (mean age of 10.2 years). Various classifications of caudal maxillectomy (+/− orbital reconstruction) were performed in all dogs via a maxillary transfacial approach (Table 1).

**Table 1:** Case information, diagnosis, surgical procedure, complications and follow-up duration.

Case	Age (yrs)	Sex	Weight (kg)	Pre-operative Diagnosis	Post-operative Diagnosis	Tumor Extent	Intended Surgical Margin (cm)	Achieved Surgical Margin	Surgical Procedure	Approach*	Complications/Revision	Follow-up Duration (mos)
1	12	FS	15.4	CAA	CAA	Left maxillary third-fourth premolar teeth	1	Clean	Caudal maxillectomy; OZMC excision; orbital reconstruction (titanium mesh)	1, a, b	Edema; rostral flap necrosis; oral dehiscence; MRSP infection; ONF revision surgery	7
2	11	MN	28.4	Poorly differentiated sarcoma	Poorly differentiated sarcoma	Right maxillary first premolar tooth-hamulus of pterygoid bone	2	Clean	Caudal maxillectomy; OZMC excision; orbital exenteration; temporalis myofascial flap	1, a, c, d	Edema; temporalis myofascial flap necrosis; ONF revision surgery	9
3	7	FS	13.1	FSA	FSA	Right maxillary second premolar tooth-OZMC	2	Clean	Caudal maxillectomy; OZMC excision; orbital exenteration; temporalis myofascial flap	1, a, c, d	Edema; oral dehiscence; ONF revision surgery; tumor recurrence; humane euthanasia	6
4	9	FS	6.3	Odontogenic cyst	Odontogenic cyst	Right maxillary second premolar tooth-OZMC	0.5	Clean	Caudal maxillectomy; OZMC excision; orbital reconstruction (titanium mesh)	2, a, b	Edema	8
5	9	FS	21.3	PGCG	OSA	Left maxillary first-second molar teeth	1	Dirty	Caudal maxillectomy	2, a	Edema; humane euthanasia	2
6	13	MN	18.0	APA	APA	Right maxillary third premolar tooth-OZMC	1	Clean	Caudal maxillectomy; OZMC excision; orbital reconstruction (titanium mesh)	2, a, b	Edema	9

CAA, canine acanthomatous ameloblastoma; FSA, fibrosarcoma; OSA, osteosarcoma; PGCG, peripheral giant cell granuloma; APA, amyloid producing ameloblastoma; *1, maxillary transfacial approach; 2, modified transfacial approach; a, subciliary extension; b, zygomatic extension; c, superciliary extension; d, temporalis extension.

### Medical records review

Medical records of the six patients were queried between January 2015 and December 2022 for the following information: history, oral examination results, diagnostic imaging and histopathologic results, surgical planning methods, surgical plan/approach and outcome. Follow-up was obtained from medical records and telephone interviews with owners. Complications were temporally categorized: intra-operative – during surgery; post-operative acute – within 7 days of surgery; post-operative chronic – >7 days after surgery.

### Imaging and surgical planning

Prior to surgery, oncologic staging and head, cervical and thoracic contrast-enhanced computed tomographic (CT; GE Lightspeed Ultra, GE Healthcare, Milwaukee, WI), imaging were performed in all cases. Aspirates of mandibular lymph nodes, followed by ultrasound guided aspirates of abnormal (as dictated by imaging results) medial retropharyngeal lymph nodes, were obtained for cytology. Surgical plans for all patients were developed with the aid of virtual surgical planning (VSP) as previously described (13). DICOM files for each patient were imported into a dedicated image segmentation and three-dimensional modeling software (Mimics 21.0, Materialise, Leuven, Belgium). A mask of the skull was created using a thresholding operation and a 3D model of the subject skull was created. Osteotomies were planned with margins appropriate for the biological behavior of the pathology in each case, delineated and performed completely in the virtual environment as part of the surgical planning/rehearsal. In some cases, multiple 3D models were exported into a standard tessellation language mesh and printed to scale.

### Anesthesia

All dogs were placed under general anesthesia for surgical excision using tailored anesthetic protocols as determined by a board-certified anesthesiologist (Table 2). Standard orotracheal intubation was performed to maintain a secure airway in all patients. Ultrasound guided trigeminal nerve blocks with 0.5% bupivacaine were performed in three patients and a maxillary nerve block was performed in three patients. The patients were positioned in lateral recumbency and sterilely prepared for surgery. A temporary ipsilateral tarsorrhaphy was performed in all patients to protect the globe or to facilitate orbital exenteration.

**Table 2:** Anesthetic and antibiotic protocols.

Case	Diagnostic Imaging	Surgical Anesthetic Protocol	Post-Operative Antibiotics
1	Butorphanol and dexmedetomidine premedication. Induction with alfaxalone	Hydromorphone and dexmedetomidine premedication. Induction with ketamine and alfaxalone	Amoxicillin/Clavulanic Acid
2	Hydromorphone and dexmedetomidine premedication. Induction with ketamine and propofol	Hydromorphone and dexmedetomidine premedication. Induction with ketamine and propofol	Amoxicillin
3	Methadone and dexmedetomidine premedication. Induction with alfaxalone	Hydromorphone and dexmedetomidine premedication. Induction with ketamine and alfaxalone	Amoxicillin
4	Hydromorphone and dexmedetomidine premedication. Induction with alfaxalone	Hydromorphone and dexmedetomidine premedication. Induction with alfaxalone	Amoxicillin/Clavulanic Acid
5	Fentanyl and dexmedetomidine premedication. Induction with ketamine and propofol	Fentanyl and dexmedetomidine premedication. Induction with ketamine and propofol	None
6	Butorphanol and dexmedetomidine premedication. Induction with propofol	Fentanyl and midazolam premedication. Induction with ketamine and alfaxalone	None

*In all cases standard drug doses and durations (antibiotics) were used.

### Surgical technique

**Figure 1:**
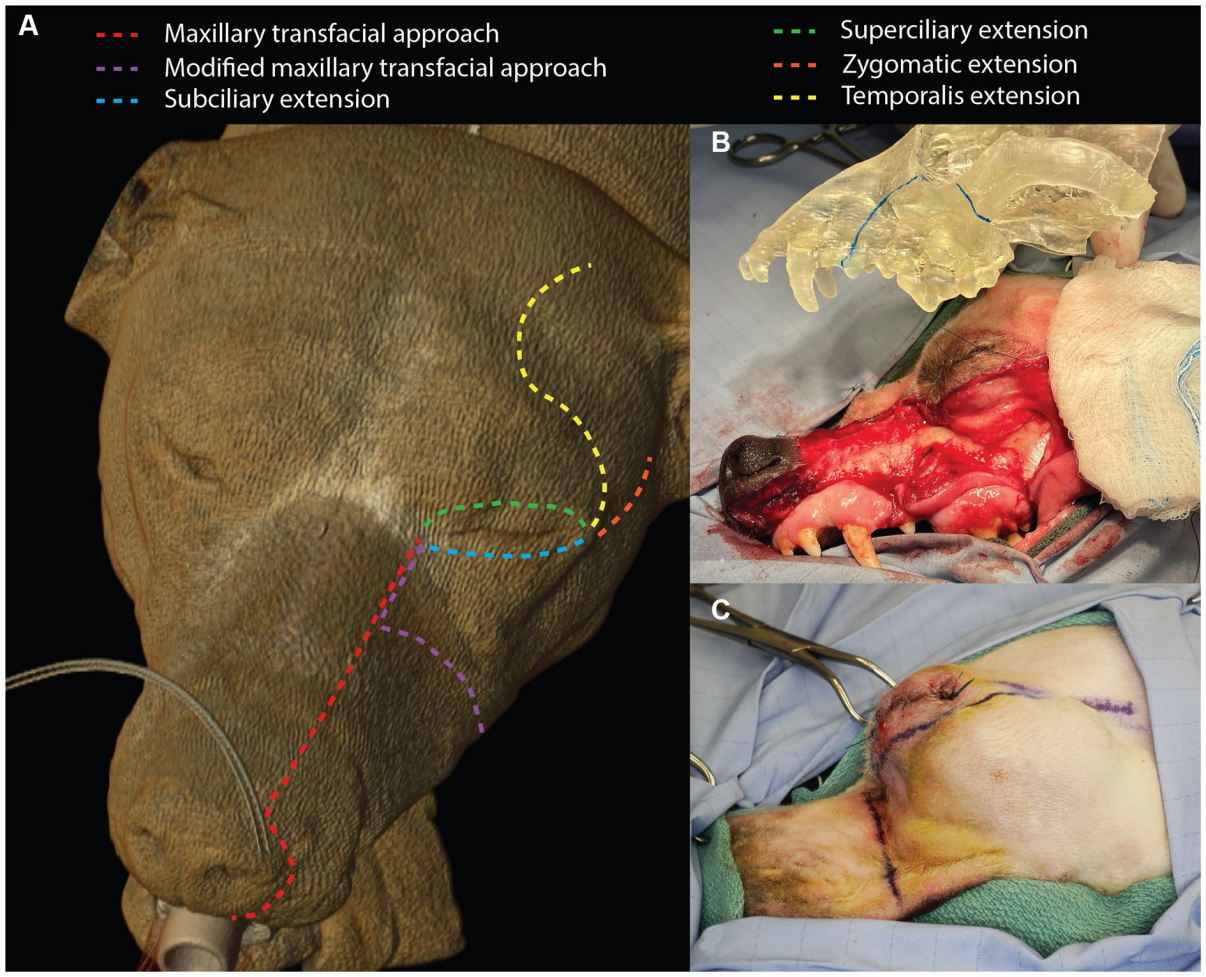
Depiction of the maxillary transfacial approach and the modified maxillary transfacial approach in the dog as well as extension incisions **(A)**. The degree of exposed maxilla and OZMC offered by the approach can be appreciated in panel **(B)**. Panel **(C)** illustrates the incision locations for the modified maxillary transfacial approach in a live patient.

A sterile surgical marker was used to delineate the cutaneous incisions and the intra-oral intended surgical margins (1–2 cm based on tumor type and biological behavior). The maxillary transfacial approach was implemented in three patients. A full-thickness labial incision was made at the philtrum that extended vertically to the nasolabial groove, around the nasal ala and superior to the dorsolateral aspect of the maxilla (Figure 1). The incision was then extended caudally to the level of the medial canthus of the ipsilateral eye where the angularis oculi vein was isolated, ligated and transected. Subciliary, superciliary, zygomatic, and temporalis extensions were used as necessary based on exposure and oral reconstruction demands (Figure 1). The oral incision was made just dorsal and parallel to the mucogingival junction up to the marked surgical margin and then followed the dorsal aspect of the surgical margin caudally (Figure 1). The infraorbital neurovascular bundle was ligated and transected at the level of the foramen. The flap, including the skin, musculature and periosteum, was raised using a periosteal elevator. This allowed for complete caudal reflection of the transfacial flap, which provided exposure to the caudal maxilla and OZMC and the ability to continue with the intra-oral approach to tumor excision. The intra-oral approach to the tumors continued along the surgical markings through the gingiva, vestibular mucosa and palatal mucosa.

In three patients, a modification to the maxillary transfacial approach (modified maxillary transfacial approach) was made to preserve the infraorbital neurovascular bundle. A full thickness transfacial incision that extended through the mucocutaneous junction of the superior labia up to the dorsolateral aspect of the maxilla was made immediately caudal to the level of the infraorbital foramen (Figure 1C). The superior labial, infraorbital, lateral nasal and dorsal nasal veins as well as the superior labial artery were ligated and transected. The dorsal buccal branch of the facial nerve was also transected. Care was taken to maintain the main branch of the facial vein with the flap. The oral mucosal incision was made dorsal to the mucogingival junction and dictated by intended surgical margins. In all cases, osteotomies were made according to the individual surgical plans with a piezosurgical unit. Subsequently, the excised tissue was removed.

**Figure 2:**
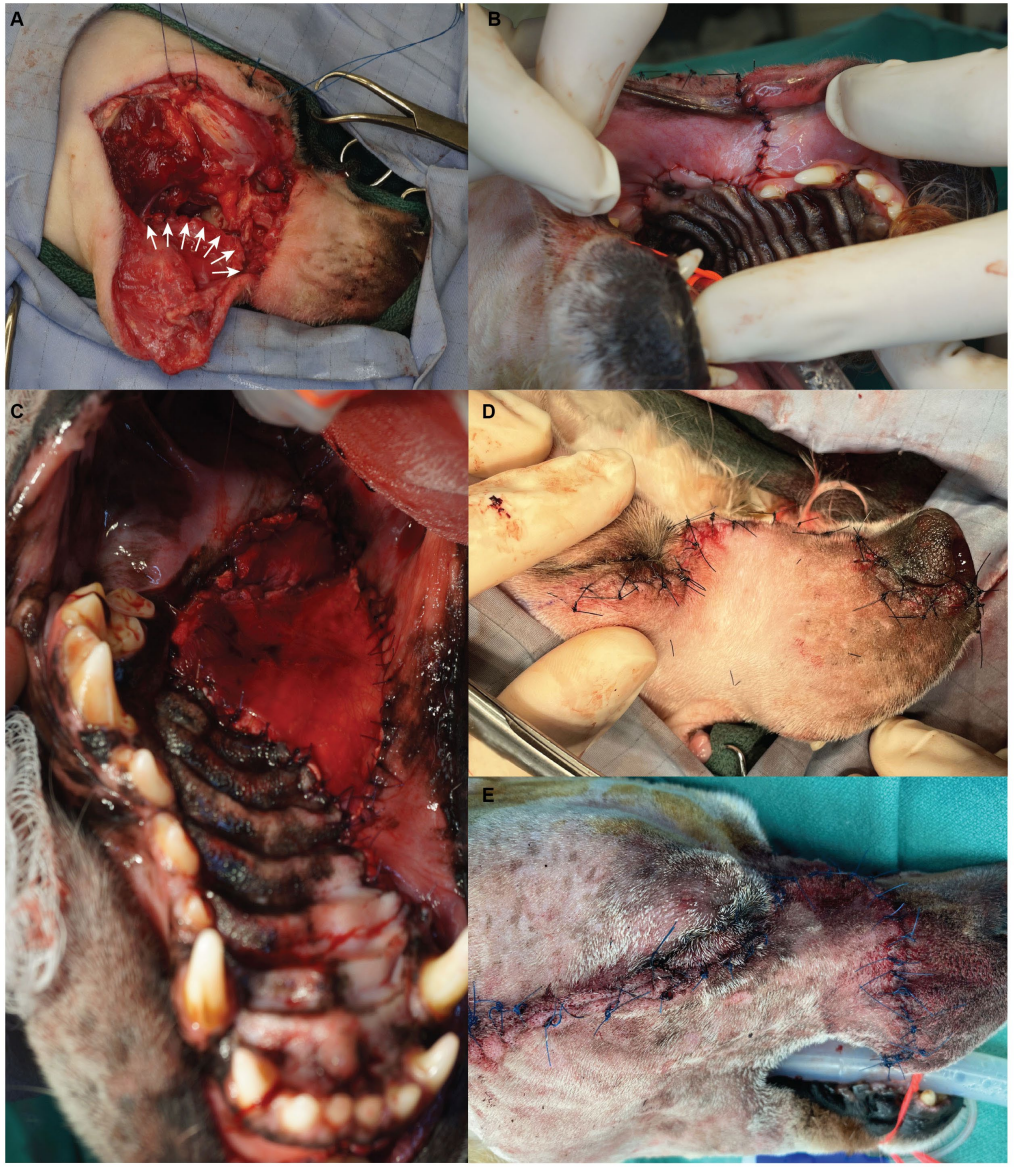
Surgical photographs depicting the flap closure sequence. The oral mucosa is sutured with a simple interrupted pattern (**A** – white arrows, **B**); if a myofascial flap is required for oral reconstruction it is sutured within the space between the vestibular mucosa and the hard palate mucoperiosteum **(C)**; after closure of musculature, the dermis is sutured in a simple interrupted pattern (**D** – maxillary transfacial approach, **E** – modified maxillary transfacial approach).

Closure of the surgical sites occurred in four layers. First, oral mucosa was separated from the overlying flap dermis via blunt dissection and subsequently sutured in a simple interrupted pattern to the hard palate mucoperiosteum (Figures 2A,B). When a temporalis myofascial flap was used for oral reconstruction, the vestibular mucosa was sutured to the fascia of the myofascial flap instead of the hard palate mucoperiosteum (Figure 2C). The orbicularis oris, buccinator, levator labii superioris, and the levator nasolabialis muscles were then apposed in a simple interrupted pattern. The submucosa and dermis were routinely closed (Figures 2D,E). All patients received a bupivacaine liposome suspension (Nocita, Elanco, Greenfield, IN) injected into the surrounding musculature, submucosal and subcutaneous tissues for sustained post-operative local analgesia at various points during the wound closure. Sentinel lymph node mapping was not performed in any case.

### Post-operative care

All dogs were recovered in the critical care unit (CCU) for administration of IV fluids, tailored pain management protocols via continuous rate infusion (CRI) and continuous pain assessment. Pain management was achieved with tailored multimodal approach in consultation with a board-certified anesthesiologist and/or criticalist. All dogs received application of a cool compress at the surgical site every 4 h until discharge from the hospital. Dogs were administered non-steroidal anti-inflammatory drugs via injection and/or mouth for 3–7 days duration. After 12–24 h in CCU, dogs were transitioned from CRI administration of opioids to either transmucosal or transdermal opioid administration. Dogs were discharged with instructions to return in 10–14 days for assessment and skin suture removal. Systemic antibiotics were not routinely prescribed (Table 2).

## Results

Lesion diagnoses based on post-operative histopathological evaluation included one poorly differentiated sarcoma, one fibrosarcoma (FSA), one canine acanthomatous ameloblastoma (CAA), one amyloid producing ameloblastoma (APA), one osteosarcoma (OSA) and one multi-loculated odontogenic cyst (Table 1). Lesions were located in the right maxilla/OZMC (4) and left maxilla/OZMC (2). The largest lesion extended from the maxillary first premolar tooth to the hamulus of the pterygoid bone. The smallest lesion extended from the maxillary third premolar tooth into the ventral orbit. Oncologic staging was considered negative in all cases.

### Surgical procedures and approach

In three cases, caudal maxillectomy with OZMC excision and orbital reconstruction (titanium mesh implant) was performed (Table 1). In these cases, the resultant oral soft tissue defect was closed with a buccal pedicle advancement flap. Caudal maxillectomy with OZMC excision and orbital exenteration was performed in two cases (Table 1). Lesions in these two cases extended to and effaced the globe. In order to achieve appropriate margins and to properly access the medial orbitotomy with an appropriate angle, orbital exenteration was deemed necessary. Both of these cases had oral soft tissue reconstruction with a temporalis myofascial axial pattern flap. One case received a caudal maxillectomy and the oral soft tissue defect was closed with a buccal pedicle advancement flap (Table 1).

The maxillary transfacial approach was performed in three cases and the modified maxillary transfacial approach was performed in three cases (Table 1). The subciliary extension was used in all cases. Superciliary extensions were used in two cases that required orbital exenteration. The temporalis extension was also used to access the temporalis muscle for oral reconstruction in these two cases. Three cases required a zygomatic extension to facilitate orbital reconstruction with a titanium mesh implant. In all cases the extra-oral aspect of the transfacial approach was closed routinely.

Surgical visualization and accessibility were deemed excellent in all cases. Appropriate angle of approach was easily achieved for the required osteotomies (14). Visualization was considered ideal for margin assessment, vessel ligation, graft harvesting and reconstruction efforts. Histologic evaluation of excision margins revealed clean margins in all but one case (case 5). In this case the pre-operative incisional biopsy was consistent with peripheral giant cell granuloma (PGCG), which is a benign but aggressive reactive lesion. However, the post-operative histopathology was consistent with osteosarcoma (OSA). Surgical revision was recommended, however, the owners elected humane euthanasia two-months after surgery.

### Complications

#### Intraoperative

No patients experienced any intra-operative complications (Table 1).

#### Postoperative acute

**Figure 3:**
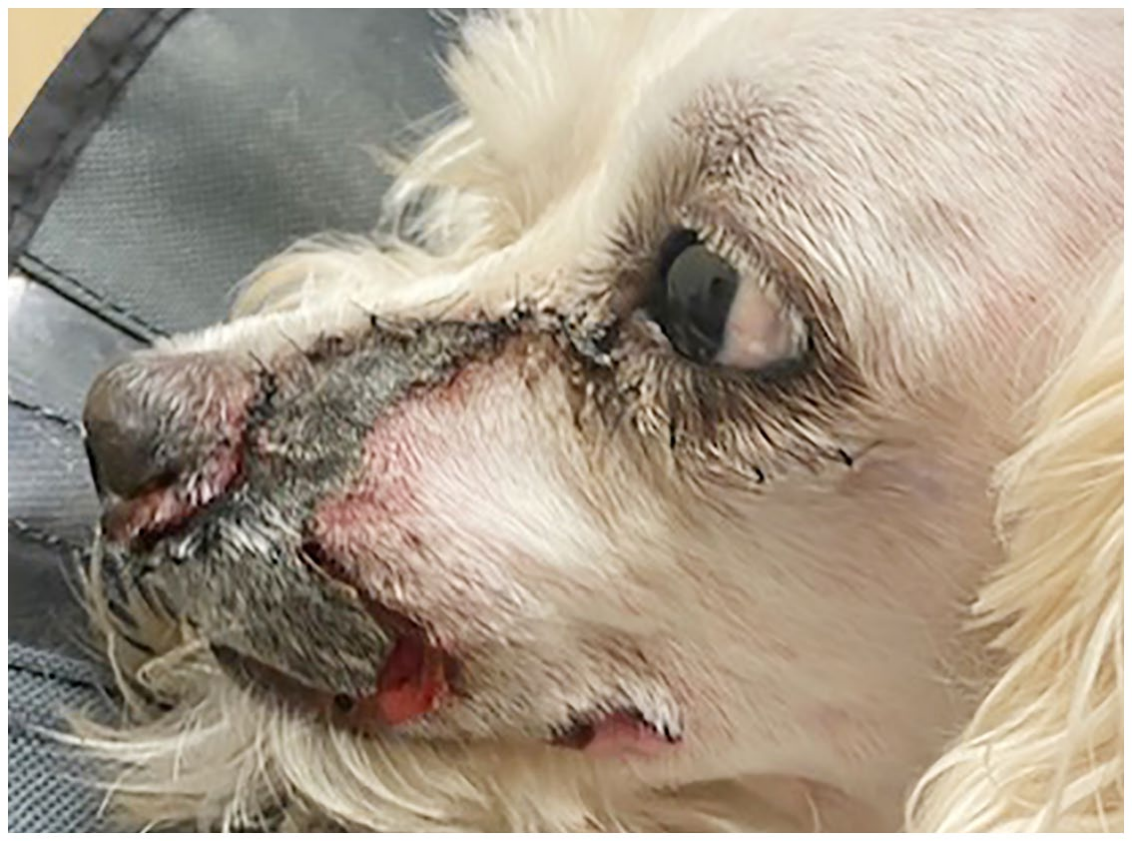
One-week postoperative photograph of case 1 in which the dorsorostral 25% of the skin flap become necrotic.

All cases experienced post-operative edema, which responded well to application of cool-compresses and resolved within 7 days. In one case that received orbital reconstruction with titanium mesh (case 1), the dorsorostral ~25% of the transfacial flap became necrotic within the first post-operative week and subsequently developed a methicillin-resistant *Staphylococcus pseudointermedius* (MRSP) infection (Figure 3). The necrotic tissue was debrided and the resultant facial defect was closed with a labial advancement flap. The MRSP infection resolved during a 14–day course of amoxicillin-clavulanate.

Oral dehiscence occurred in the two cases that required oral soft tissue closure with temporalis myofascial axial pattern flaps. One of these cases (case 2) was secondary to temporalis myofascial flap necrosis. In this case, the resultant defect was subsequently closed with an angularis oris axial pattern flap. In the other case (case 3), the resultant defect was closed 6 weeks after dehiscence with a buccal pedicle flap advanced to the surviving myofascial flap. Tumor recurrence was noted 5 months post-operatively and the patient was humanely euthanized 1 month later.

#### Postoperative chronic

In case 1, a 1 × 1 cm oronasal defect developed 6 weeks post-operatively. It was determined that the underlying titanium mesh was contributing to the defect. In a subsequent surgery, the titanium mesh was recontoured to prevent contact with a labial advancement flap that was used to close the oral defect and healed without complication.

## Discussion

The surgical approach described here is based on the Weber–Ferguson approach, which is widely accepted as the best approach to the midface and retromaxillary region in the human patient where enhanced exposure is required (7, 8). The surgical anatomy of caudal oromaxillofacial tumors in dogs presents a challenge. Previously published manuscripts describing approaches in dogs demonstrated the benefit of greater access and exposure to the surgical anatomy (1–3). Combined intraoral and extraoral approaches may require the patient to be repositioned throughout the surgical procedure. The maxillary transfacial approach described here presents an alternative approach to the same anatomic region with greater exposure. In addition, the approach facilitates large orbital and oral reconstruction efforts, while maintaining the patient in lateral recumbency throughout the procedure. Unlike a commissurotomy, the maxillary transfacial approach avoids damage to the angularis oris cutaneous artery. By maintaining this cutaneous artery, the option to perform an angularis oris axial pattern flap is retained, should this be needed for revision surgery or additional repair as noted in one of the cases presented here.

The traditional Weber–Ferguson approach in the human patient incorporates an ascending incision through the philtrum. The placement of the incision is chosen primarily for esthetic reasons and is also a practical anatomical choice (5). As the approach is extended caudally, the infraorbital neurovascular bundle may be preserved by performing an infraorbital orbitotomy (15). Preservation of the infraorbital neurovascular bundle was achieved in three of our patients by using a modified maxillary transfacial approach with the rostral aspect of the incision located immediately caudal to the infraorbital foramen. Given that esthetics is a lower priority consideration in veterinary medicine and scar tissues are typically obscured by hair regrowth, this an acceptable approach in dogs. In addition, placing the rostral incision in the philtrum of dogs contributes to a long and relatively narrow flap and could place the rostral extent of the flap at risk for ischemic necrosis. It is possible this was the cause for the rostral cutaneous necrosis experienced in one of our patients. However, the necrosis pattern in that patient may also be consistent with loss of arterial blood flow in the rostral dorsal nasal artery (a branch of the infraorbital artery) secondary to surgical ligation, rather than flap design/morphology.

The subciliary and zygomatic extensions were used in six and three patients, respectively. These extensions provided the surgeons with excellent exposure to the ventral orbit and zygomatic arch for precise osteotomies and subsequent orbital reconstruction. The temporalis extension was employed in two patients, which allowed exposure to the temporal region and use of a temporalis myofascial flap to reconstruct large oral defects following tumor resection. The superciliary extension was utilized in two patients that underwent a resultant transpalpebral orbital exenteration.

The most common reported intra-operative complication in maxillectomy procedures is excessive hemorrhage (16). Intra-operative complications, including excessive hemorrhage, were not experienced in any cases. We attribute minimal blood loss despite the large excision size in some cases to meticulous hemorrhage control and the use of a piezosurgical saw (17). Due to the mechanism of action of piezosurgical saws, soft tissues including large blood vessels, are not incised, resulting in minimal blood loss.

The most common acute post-operative complication, seen to some degree in all cases presented here was flap edema. The flap edema was likely the result of the transection of several vessels including the superior labial artery and vein, the infraorbital vein and the angularis oculi vein as well as their associated lymphatic vessels. The edema responded well to cool-compresses and rapid resolution and may be at least partly attributed to the vast anastomosis and arborization of vessels forming a rich vascular network within the flap.

Intra-oral dehiscence occurred in three (50%) of the cases. All of these cases (cases 1–3) were very large oral defects. Two cases (cases 2, 3) required a temporalis myofascial flap to reconstruct the oral mucosa. In one of these cases, the flap became necrotic presumably from strangulation of the superficial temporal artery. We believe the large size of these defects and tension and/or vascular compromise was the primary cause of wound dehiscence (18). In the third case, the cause of dehiscence was determined to be contact from the titanium mesh used to reconstruct the orbit. The intra-oral wound healed after mesh recontouring and simple closure. It does not appear that the design of the maxillary transfacial approach contributed to intra-oral wound dehiscence in any of these cases. However, we cannot definitively exclude the possibility that flap edema and/or flap design may have contributed, at least in part, to the wound dehiscence in these three cases. A larger sample size and clinical experience with this approach is needed to fully elucidate this question.

The maxillary transfacial approaches described here provided exceptional access and visibility to the caudal oral cavity and OZMC. Compared to traditional oral and combined intraoral/extraoral approaches, the maxillary transfacial approach described here provides enhanced exposure to facilitate wide lesion excision with soft and hard tissue reconstruction efforts. In addition, avoiding commissurotomy preserves the angularis oris artery, which may be used for future reconstruction efforts should it be needed. Combined with thoughtful surgical planning and sound surgical principles, the maxillary transfacial approach appears to be a useful technique in complex oromaxillofacial pathology excision and reconstruction. Larger studies evaluating the surgical outcomes in patients that undergo maxillary transfacial approaches compared to traditional approaches are warranted.

## Data availability statement

The original contributions presented in the study are included in the article/supplementary material, further inquiries can be directed to the corresponding author.

## Author contributions

GT: Conceptualization, Formal analysis, Investigation, Methodology, Visualization, Writing – original draft, Writing – review & editing. MC: Investigation, Writing – original draft. JS: Conceptualization, Data curation, Formal analysis, Funding acquisition, Investigation, Methodology, Project administration, Supervision, Visualization, Writing – original draft, Writing – review & editing.
